# Milk kinship hypothesis in light of epigenetic knowledge

**DOI:** 10.1186/1868-7083-4-14

**Published:** 2012-09-18

**Authors:** Hasan Ozkan, Funda Tuzun, Abdullah Kumral, Nuray Duman

**Affiliations:** 1 Department of Pediatrics, Division of Neonatology, Dokuz Eylul University Faculty of Medicine, Mithatpasa Street, Inciralti, 35340, Izmir, Turkey

**Keywords:** Breast milk, Milk banking, Wet nurse, Epigenetics, Stem cells, microRNA, Inheritance

## Abstract

**Background:**

A wet nurse can be used if a baby’s natural mother is unable or chooses not to breastfeed her infant. The practice of using wet nurses is ancient and common to many cultures.

**Presentation of the hypothesis:**

We hypothesize that infants breastfeeding from the same woman may develop consanguinity even in cases in which they are not blood relatives, and that children of two individuals breastfed by the same woman may thus be at risk of several genetic diseases because of such consanguinity.

**Testing the hypothesis:**

Possible evidence for the milk kinship hypothesis is to be found in the composition of breast milk, which is composed of living substances such as stem cells or substances that can affect epigenetic regulation such as microRNAs.

**Implications of the hypothesis:**

If these epigenetic modifications are heritable, marriages between individuals breastfed by the same woman may result in the same consequences as consanguineous marriages. In this paper, we attempt to assess this possibility.

## Background

A wet nurse can be used if a baby’s natural mother is unable or chooses not to breastfeed her infant. The practice of using wet nurses is ancient and common to many cultures. After the Industrial Revolution, when wet nurses became difficult to find, human milk banking began. In Islamic law, breastfeeding institutes a form of kinship relation (milk kinship). This has historically been a medium for complex social and political networks in the Middle East. As explained in the Qur’an (An-Nisa Surah), a child who has nursed from a woman becomes a relative of the nursing woman, and also a milk sibling to others who have shared her breast, a relationship that prevents future marriage. However, the Qur’an does not explain why such marriages are forbidden.

## Presentation of the hypothesis

We have hypothesized that breastfeeding from the same woman may lead to consanguinity between individuals even with no blood relation, and that children born of a marriage between two such individuals may thus be at risk of certain genetic diseases as a result of this consanguinity. In this paper, we attempt to identify the possible reasons for this proposal according to current scientific knowledge.

## Testing the hypothesis

Breastfeeding provides multiple lifelong biological advantages to children, including increased survival and improved neurocognitive and immune functioning. These and other advantages have been linked to breast milk components, including fatty acids [1], growth factors [2], immunological factors [3,4], live maternal cells [5,6] and some genetic materials [7,8]. Possible evidence for the milk kinship hypothesis is to be found in the composition of the breast milk, which is a living substance that includes i) genetic materials (such as microRNAs), ii) stem cells and iii) organic substances affecting epigenetic regulation mechanisms and gene transcription.

Human milk samples contain exosomes, which are tiny endosome-derived membrane vesicles (approximately 30 to 100 nm in diameter) that are released into the extracellular environment from a variety of different cells [9]. Exosomes are present in various body fluids (such as amniotic fluid, blood, saliva and urine) and contain distinct subsets of microRNA (miRNA) [10]. One of the most significant factors in support of the present hypothesis is the presence of miRNAs in breast milk. miRNAs are small regulatory RNA molecules that modulate the activity of specific miRNA targets and play important roles in a wide range of physiological and pathological processes, being involved primarily in the negative regulation of gene expression [11,12]. Recent studies have demonstrated that miRNAs control the expression of important epigenetic regulators, including DNA methyltransferases and histone deacetylases. Taken together, post-transcriptional regulation by miRNAs and transcriptional control machinery by epigenetics cooperate with each other to organize the whole gene expression profile and to maintain physiological functions in cells. Once this miRNA-epigenetics regulatory circuit is disrupted, normal physiological functions are interfered with, contributing to various disease processes [13]. These miRNAs may function as secreted signaling molecules to influence the recipient cell phenotypes [14,15].

Because immune-related miRNAs are well studied and their functions are clarified, studies have focused on their presence in human breast milk. Kosaka *et al*. profiled miRNA expression in human breast milk and detected high expression levels of immune-related miRNAs (miR-181a, miR-17, miR-155, miR-150, miR-223) in the first six months of lactation. Many different types of miRNAs in human breast milk were detected in this study, related to liver, nervous system and pancreatic tissues; however, the functions of many of these are still unknown [7]. Weber *et al*. examined the spectrum of miRNA in various extracellular fluids including human milk. The top twenty most abundant miRNAs in breast milk were the following: miR-335, miR-26a-2, miR-181d, miR-509-5p, miR-524-5p, miR-137, miR-26a-1, miR-596, miR-580, miR-130a, miR-515-3p, miR-513c, miR-671-5p, miR-490-5p, miR-367, miR-181b, miR-598, miR-515-5p, miR-578, miR-487b. The miRNA species that were uniquely detected in breast milk were the following: miR-193b, miR-10a, miR28-5p, miR-924, miR-150, miR-518c and miR-217 [10]. Recently, a study by Zhou *et al*. has provided evidence that the immune-related miRNAs (miR-148a-3p; miR-30b-5p; miR-182-5p; and miR-200a-3p) were enriched in breast milk exosomes [16]. Both studies demonstrated that exosomal miRNAs are resistant to general harsh conditions, such as prolonged room temperature incubation, very acidic conditions, RNase digestion and even boiling, indicating that breast milk allows dietary intake of miRNAs by infants [7,16]. These studies clearly suggest that miRNA is a genetic material admitting of transfer from mother to infant, and the transfer of genetic material as miRNA from human to human occurs by means other than through sexual reproduction.

The presence of stem cells in breast milk is another important factor supporting the milk-kinship hypothesis. The presence of low numbers of maternal cells or DNA in offspring is known as maternal-fetal microchimerism. Breast milk is an important source of microchimerism in which trafficking of cells from one individual to another occurs, and cells of maternal origin persist in offspring for years [17]. In an experiment in which mice were fed with milk containing marked maternal cells, the marked cells were demonstrated to invade the tissues of offspring [18]. Stem cell self-renewal and differentiation also interacts dynamically with epigenetic mechanisms. Some miRNAs are specifically expressed in stem cells, controlling stem cell self-renewal and differentiation by negatively regulating the expression of certain key genes in stem cells [19].

In general, these epigenetic regulations are cleared and re-established each generation, but there have been reports in a number of model organisms that at some loci in the genome this clearing is incomplete. Recent findings support the contention that epigenetic mechanisms may also be meiotically heritable, described as transgenerational epigenetic inheritance, and in this way an environmental event in one generation could affect the phenotype in subsequent generations [20-22]. Inheritance of epigenetic variations may account for a significant part of heritability in human and in mammalian models. Heritable epigenetic variations were reported in plants under the name ‘paramutation’ more than 50 years ago. Paramutation describes the transfer of an acquired epigenetic state to an unlinked homologous locus, resulting in a meiotically heritable alteration in gene expression. Numerous examples of paramutation and paramutation-like phenomena have now emerged, with evidence that implicates small RNAs in the transfer and maintenance of epigenetic states [23]. Rassoulzadegan and colleagues provided clear evidence of heritable epigenetic modification (paramutation) of the Kit gene of the mouse and strong evidence that RNA was involved [24]. Later, the same study group illustrated the relevance of paramutation to pathophysiology by injecting fertilized mouse eggs with miRNAs (miR-1) targeting cyclin-dependent kinase 9 (Cdk9), a key regulator of cardiac growth. Their results revealed inheritance of epigenetic cardiac hypertrophy over at least three generations, and efficient hereditary transmission was correlated with the presence of miR-1 in the sperm nucleus. Based on these results, the mouse models suggest the possible establishment and familial transmission of epigenetic changes with pathological consequences [25].

It is important to determine the windows of greatest susceptibility to these epigenetic modifiers. It is possible that the period in which the individual is most vulnerable to these changes is before the age of two, during the suckling period. In other words, such a relationship cannot be established if the milk recipient has already completed his or her developmental period. Possible explanations for this phenomenon, in light of current knowledge, may be: i) inadequacy of the child’s immune system in the early infancy period to reject maternal living cells and genetic materials, ii) increased plasticity of the human body during the early developmental period, and iii) increased vulnerability of the epigenome to epigenetic changes during the early developmental period.

## Implications of the hypothesis

In conclusion, even though the fate of the nursing infant in terms of the biologically active materials such as stem cells and miRNAs is not entirely understood, current knowledge indicates that these materials can invade the tissue of the infant, especially in the early developmental period [7]. They can effect epigenetic remodeling by interacting with epigenetic regulatory mechanisms in different ways.

As a result, new phenotypes or diseases may occur according to the affected gene expression. If such epigenetic changes induced by breast milk can be heritable, the new epigenotypes will be expressed in later generations. So, if infants are fed by the same mother regularly, they may share similar epigenotypes. If these epigenetic modifications can be heritable to the next generations, marriages between individuals breastfed by the same woman may result in the same consequences as consanguineous marriages.

Anecdotally, it was believed that the wet nurse’s milk carried all her physical and mental qualities, her emotions, her food and drink, and her diseases. However, to our knowledge there is no scientific proof supporting this common belief.

Understanding the biological role of the genetic materials transmitted to the nursing infant and the establishment of experimental models may assist in further explaining this mysterious phenomenon.

## Competing interests

The authors declare that they have no competing interests.

## Authors’ contributions

HO stated the original hypothesis and helped to draft the manuscript. FT improved the hypothesis and drafted the manuscript. AK participated in the literature search. ND participated in the literature search. All authors read and approved the final manuscript.
